# Sustainable Calcite Scale Inhibitors via Oxidation of Lignosulfonates

**DOI:** 10.1021/acsomega.4c02716

**Published:** 2024-05-30

**Authors:** Sumit Ganguly, Malcolm A. Kelland, Ross J. Ellis, Martin Andresen, Sreedhar Subramanian, Aspasia Theodossiou

**Affiliations:** † Department of Chemistry, Bioscience and Environmental Engineering, Faculty of Science and Technology, University of Stavanger, N-4036 Stavanger, Norway; ‡ Borregaard AS, Hjalmar Wessels vei 6, 1721 Sarpsborg, Norway

## Abstract

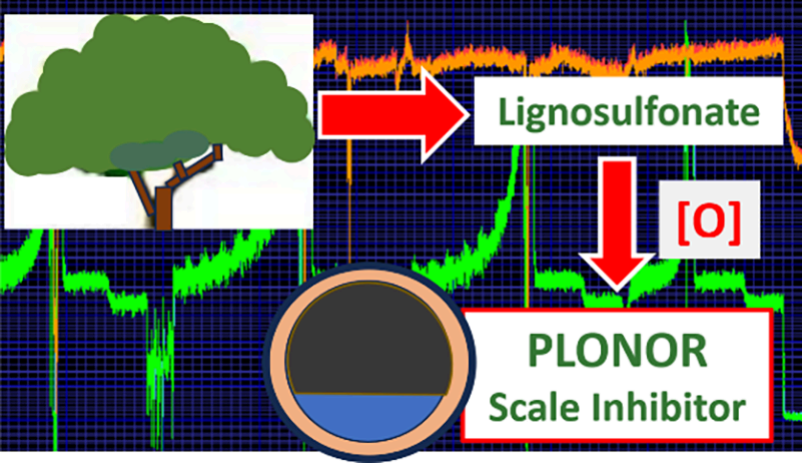

Deposition of inorganic scales in wells, flow lines, and equipment is a major problem in the water treatment, geothermal, or upstream oil and gas industries. Deployment of scale inhibitors has been adopted worldwide for oilfield scale prevention. Commercial synthetic scale inhibitors such as polymeric carboxylates and sulfonates or nonpolymeric phosphonates offer good scale inhibition performance but often suffer from one or more limitations including biodegradability, calcium compatibility, and thermal stability. Lignin-based biomaterials such as sodium lignosulfonates are natural, sustainable, and widely available polymers that are accepted for use in environmentally sensitive areas. Here we show that, although lignosulfonates perform relatively poorly as calcite scale inhibitors in dynamic tube blocking tests, oxidized lignosulfonates show a much improved inhibition effect by a factor of 20-fold. The oxidized lignosulfonates are easy to prepare in a 1-step reaction and show excellent calcium compatibility and thermal stability, useful for downhole squeeze treatments in high temperature wells. This present study unequivocally establishes oxidized lignosulfonates as a new class of sustainable green scale inhibitors, thereby bridging the gap between materials derived directly from nature and the classic synthetic polymeric scale inhibitors.

## 1 Introduction

Scaling is a major flow assurance problem in the water treatment industry and geothermal industry or during upstream hydrocarbon production.^1,2^ Scales are insoluble mineral salts originating from the aqueous supersaturated produced fluids and can start growing on any surface including wells, flow lines, process equipment, etc. If left untreated, the deposited layer will restrict the flow of fluids. Calcium carbonate (calcite) is one of the most common types of inorganic scales. Calcite scale formation is dependent on the temperature and pressure in the conduit as well as on the pH of the produced fluids and thus can be quite challenging to deal with.

Scale inhibitors (SIs) are used to prevent nucleation and/or crystal growth of the deposited scales in well and flow lines.^3−12^ Scale inhibitors are water-soluble polymeric compounds containing mainly carboxylate or sulfonate groups or nonpolymeric small molecules with phosphonate groups. Some of the commercial scale inhibitors used widely to inhibit calcite scaling are poly(acrylic acid) (PAA), poly(vinylsulfonic acid) (PVS), copolymers of maleic and acrylic acid (*co-*MA:AA), and monomeric phosphonate amines such as aminotris(methylenephosphonate) (ATMP). These scale inhibitors may be good for preventing calcite scale deposition, but they have poor biodegradability, making them not environmentally friendly. This makes it challenging to use these chemicals in areas such as offshore Norway, where strict environmental regulations are being followed. According to the Oslo–Paris (OSPAR) commission, chemicals listed as PLONOR (PLONOR = Pose Little Or NO Risk) are extremely safe for use and discharge in offshore Norway and pose no threat for aquatic marine life.^13^ There is also no limit to the volume of a PLONOR chemical that can be used offshore. For a chemical to be PLONOR listed, it should acquire a low degree of bioaccumulation, low toxicity level, and biodegradability.

Table 1 shows a list of some common commercial scale inhibitors, color coded based on their inhibition performance and other important properties.^1^ FIC is the fail inhibitor concentration, from high pressure tube blocking tests (see Experimental Section for more details). Polyacrylate and polyvinylsulfonate are petrochemical-based products and have poor biodegradability. Organophosphonates are often not desirable either, due to many exhibiting limited biodegradability and contributing to eutrophication. Additionally, many polycarboxylate and phosphonate chemicals are known to suffer from poor tolerance toward high concentrations of Ca ions present in brines and can result in depositing as an insoluble Ca^2+^–SI complex. A “green” scale inhibitor like carboxymethyl inulin (CMI) or polyaspartate (PAsp) has better biodegradability than others but has other shortcomings, particularly limited thermal stability. These inhibitors therefore cannot be used at high-temperature reservoir conditions for squeeze treatment in wells.

**Table 1:**
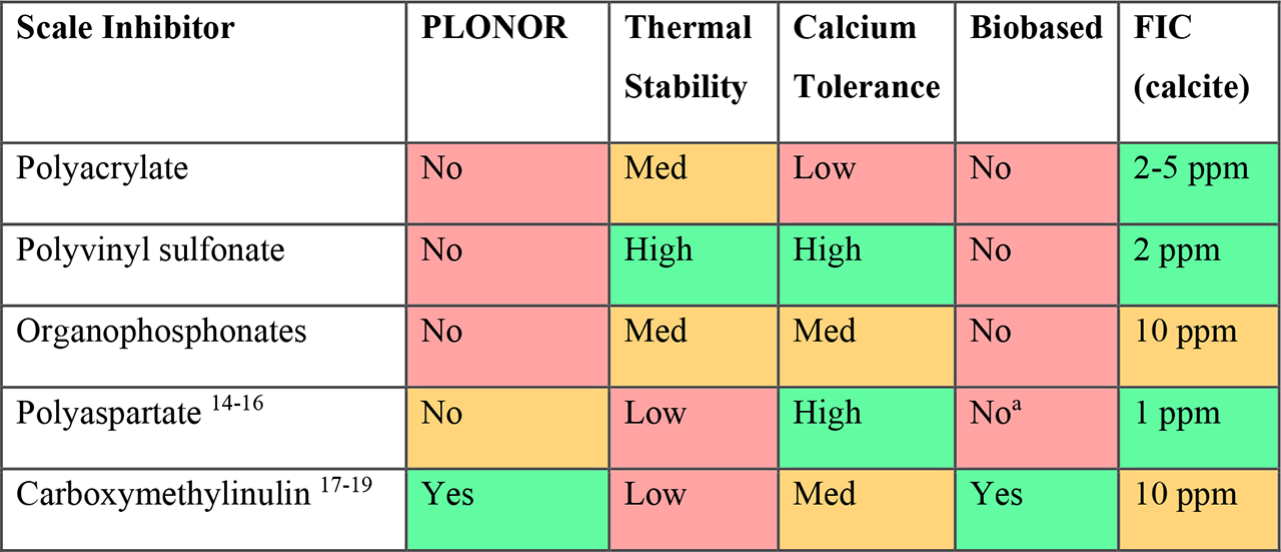
List of Some Commonly Available Calcite Scale Inhibitors (14−19)

a Aspartic acid used in some processes is produced by fermentation.

**Figure 1:**
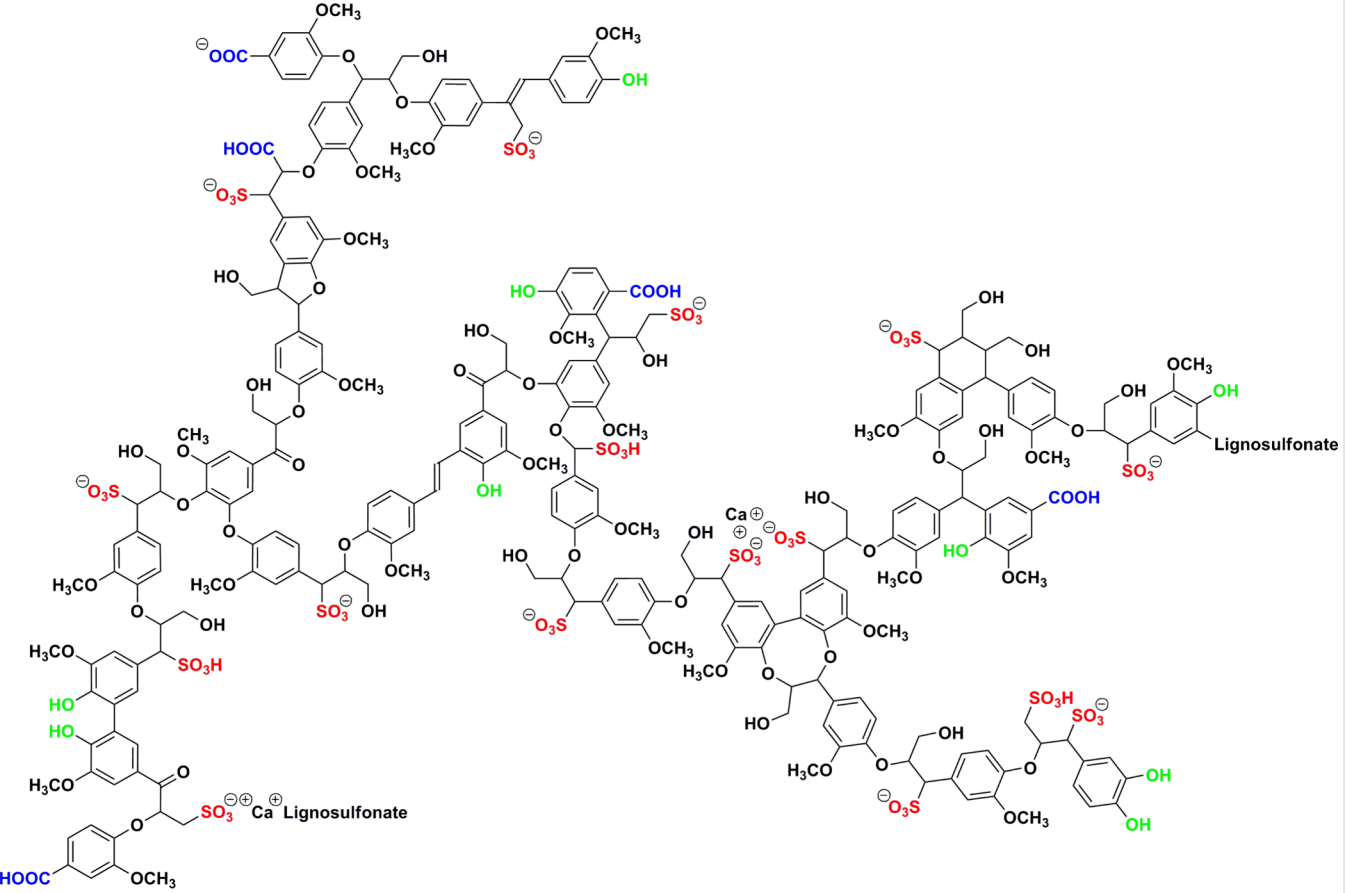
Typical structure of lignosulfonate.

There is no effective scale inhibitor as such in the current market that meets all the requirements of being PLONOR-listed, thermally stable to high temperatures, high calcium tolerant, and more importantly derived from sustainable natural raw material. In that context, we envisaged modifying lignosulfonate to make it a high-performing scale inhibitor. Sodium lignosulfonates are of interest as they are already PLONOR-listed, biobased, and water-soluble and have carboxylate and sulfonate groups that are the essential functional groups for a SI^20−23^ (Figure 1). There are already a couple of reports where lignosulfonates were used as a scale inhibitor, but they performed poorly and often involve grafting with synthetic petrochemical adducts that can compromise the PLONOR status.^24^

In this study, the oxidation route was adopted to modify the lignosulfonates without introducing foreign synthetic components to the product. Lignin ring oxidation is a well-known reaction in the pulping industry and usually is performed by oxidants like hydrogen peroxide, nitric acid, etc.^25,26^ In this study, lignosulfonates were oxidized by peracetic acid.^27^

## 2 Experimental Section

### 2.1 Materials

Sodium lignosulfonate (LS) raw material was supplied by Borregaard, Norway. Hydrogen peroxide (AnalaR NORMAPUR, 30% w/w solution) and acetic acid (glacial, >98%) were purchased from VWR and used as received. The commercial scale inhibitor benchmark MA:AA copolymer was obtained from BASF.

### 2.2 Oxidation with Peracetic Acid

About 11 g of LS (32.9% active) was placed in a 100 mL flask, and to it was added an extra 3.1 g of water to make a final 25.6% (w/w) solution. The solution was stirred at 71 °C for 10 min. A peracetic acid solution was prepared by mixing 3.65 g of H_2_O_2_ as 30% solution (amount of H_2_O_2_ taken = 30% w/w according to solid lignin mass) and 0.825 mL of glacial AcOH (ratio of H_2_O_2_:AcOH = 4:1 v/v) and ∼2 mL of 50% H_2_SO_4_. The mixture was kept in the fridge at ca. 4 °C overnight. The peracetic acid solution was added dropwise to LS solution (pH adjusted to 2) heated at 71 °C, sequentially in six or seven stages, and the solution was left to stir for approximately 30–45 min in between. In total, peracetic acid was added over a period of 5 h, and after the last addition, the solution was left to stir for an additional 3–4 h at the current reaction temperature. After a total of 10–12 h, the reaction was stopped; the solution was cooled and evaporated to dryness; and solid mass was collected.

### 2.3 Characterization of Oxidized Lignosulfonates

#### Organic Sulfur Analysis

The amount of “organic” sulfur (org. S), i.e., the amount of sulfur which is associated with the sulfonate groups attached to the lignin, i.e., the organic sulfur, is determined based on the difference between total sulfur %S(tot.) and the inorganic sulfur %S(inorg.) using the following relation:
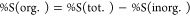
Total sulfur is determined with an element analyzer, for instance, a ThermoQuest NCS 2500. Appropriate sample amounts (for instance, 1–2 mg) are placed in tin capsules with a suitable catalyst (for instance, vanadium pentoxide). Total sulfur in the sample is then quantified using the 2,5-bis(5-*tert*-butyl-2-benzo-oxazol-2-yl)thiophene (BBOT) standard or other suitable sulfur standards. The samples are combusted at 1400 °C, and all sulfur is oxidized to SO_2_ and quantified. Inorganic sulfur is determined by measuring sulfate in oxidized samples using ion chromatography with conductivity detection (Dionex instrument using an IonPac AS11-HC column with a 13 mM OH– eluent). Samples of 30 mg are weighed into 50 mL volumetric flasks. Amounts of 10 mL of 0.5% NaOH and 5 mL of 3% H_2_O_2_ are added to oxidize sulfurous inorganic anions into sulfate. Samples are then left 12–16 h to give time to react. Milli-Q water is added and pH neutralized by adding 2 mL of 5% CH_3_COOH and diluted to the mark with Milli-Q water. Sulfate standards are prepared between 5 mg/L and 80 mg/L. The sulfate content in the oxidized samples is then determined using ion chromatography according to the instrument manual.

#### Methoxy Group Analysis

Around 30 mg of scale inhibitor was dissolved in 1000 mg of deuterated methanol (MeOD-*d*_4_), and then Amberlite IR-120 resin was added. The solution is left to stir for at least 30 min, and then 620 μL is transferred to a NMR tube with an automatic pipet. The sample is prepared twice (two parallels, and each parallel is run separately, and the average result is the final answer as the MeO content). A heteronuclear single quantum coherence spectroscopy (HSQC) experiment is then performed, where the methoxy content is calculated against a linearity curve made using an internal methoxy standard. NMR experiments were performed on a Bruker Avance III 500 MHz spectrometer using a selective inverse (SEI) probe for maximum ^1^H sensitivity. All spectra were recorded in MeOH-*d*_4_ at 300 K. ^1^H-^13^C HSQC spectra were optimized for the methoxy groups (^1^*J*_C,H_ coupling constant of 145 Hz) and recorded in the phase-sensitive mode using echo-anti-echo with the standard Bruker pulse sequence. 200 *t*_1_ 25 experiments of 1k real data points (24 scans and 16 dummy scans) were recorded with a relaxation delay of 3 s, spectral width of 9 ppm for protons, and 130 ppm for carbon. The total experiment time was 4.5 h. A squared sine window function was employed in both directions after zerofilling to a matrix of 1k × 1k data points. The spectra were phase corrected in the F2 direction, baseline-corrected in both F1 and F2 directions using the automatic fifth degree polynominal function, and the sum projection calculated for the F1 direction. The number of methoxy groups was determined from the signal intensity in the 53–58 ppm region.

#### COOH Determination Using ^31^P NMR

Characterizing the molecular structure of lignosulfonates is generally challenging due to unknown impurities that occur in lignosulfonates. Specifically, low MW carboxylic acids such as formic and acetic acids, which are formed from wood sugars and are often present in lignosulfonates, can perturb the COOH measurements. We adapted a phosphorus NMR (P NMR) method developed originally by Argyropoulos to quantify the density of COOH groups on the lignin backbone.^28^ This involves combining a purification step to remove low MW carboxylic acid impurities (e.g., formic and acetic acid) and then measuring the COOH group density on the lignin polymer using a phosphorylation reagent.

Chemicals:

- Internal Standard: Cholesterol 99%
- Phosphorylation reagent: 2-Chloro-4,4,5,5-tetra-methyl-1,3,2-di-oxa-phospholane 95%
- Deuterated solvent: CDCl_3_
- Solvents: Pyridine Anhydrous 99.8% and *N*,*N*-Dimethylformamide anhydrous 99.8%
- Drying agent: Molecular sieves 13X, Beads, 8–12 mesh
- Resin: Amberlite IR120 H+ form

The analysis of the sample takes place over 2 days:

**Day 1.** A glass pipet is filled with Amberlite. The resin is then rinsed with deionized water twice by eluting 2 × 1 mL through the pipet. The sample (200 mg) is weighed in a glass vial followed by deionized water (3 mL), and then it is left to stir for ca. 10 min. The resulting solution is then filtered through the Amberlite-filled glass pipet, and the filtrate is collected in a round glass flask. The resin is then rinsed with water twice with 2 × 1 mL and collected in the flask. The filtered sample is then frozen and freeze-dried overnight. The phosphorylation reaction is highly sensitive to water, and all reagents and components have to be dry. Hamilton syringes are used for the addition of the solvents in the reaction mixture and, therefore, have to be completely dry. In a glass beaker, molecular sieves are washed in pure acetone and then dried in an oven (105 °C) overnight. They are used for the drying of the solvents on day 2.

**Day 2.** A solvent mix of *N*,*N*-dimethylformamide (DMF) with pyridine (1:1) is made in a glass vial containing dried molecular sieves. The vial is sealed to avoid humidity. Pyridine is also added in another glass vial containing dried sieves, and the vial is sealed. This was used to make a solution of 40 mg/mL cholesterol (internal standard) in dried pyridine. A dry solution of the deuterated solvent CDCl_3_ is also prepared by adding the deuterated chloroform in a glass vial with molecular sieves and the vial sealed. Then, in a 2 mL glass vial, the dry CDCl_3_ (400 μL) was transferred followed by the phosphorylation reagent 2-chloro-4,4,5,5-tetra-methyl-1,3,2-di-oxa-phospholane (100 μL). The freeze-dried sample (30 mg ± 3 mg) was added in a 2 mL glass vial, followed by a magnetic stirrer and the solvent mixture of DMF:pyridine (1:1) (100 μL), and the mixture was then left to stir for 30 min. Next, the cholesterol solution (100 μL) was added to the reaction mixture and left to stir for 15–30 min more. Finally, the freshly made solution of the derivatization reagent in CDCl_3_ (500 μL) was added dropwise, and the reaction mixture was left to stir for 1 h prior to NMR analysis.

The ^31^P NMR experiment was run with the BBO probe at 300 K. The NMR experiment settings for a 500 MHz Avance III Bruker instrument are as follows:

**Parameters****Value**P-31 frequency202.4867 HzPulse programzgigRelaxation delay15 sNumber of scans128Number of dummy scans4Sweep width70 ppmOffset150 ppmAcquisition time1.15 sFID resolution0.43 HzTemperature300 K

Once the FID (Free Induction Decay) is acquired and Fourier transformed, the phase of the frequency domain spectrum is corrected manually (.ph) followed by apodization Fourier transformation-automatic phase correction (efp) and finally base correction (abs). Calibration of the spectrum is done by using the sharp peak of the reaction’s byproduct between the phosphorylation reagent and water at 132.20 ppm. The cholesterol’s peak at 144.8 ppm is integrated first between 145.0 and 144.4 ppm and calibrated at 1. The other signals are then integrated as follows:

**Chemical shift δ areas
(ppm)****OH type groups**150.8–145.0Aliphatic144.3–140.2Condensed phenolic140.2–138.4Guaiacyl phenolic138.4–136.9*p*-Hydroxyl phenolic135.6–133.7Carboxylic acid

The concentration (mmol of OH/g of sample) of each type of OH group is determined by following the formula:
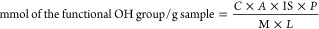
where *C* is concentration of the internal standard (mg/mL); *A* is the area of the functional OH group (when the integral of the cholesterol peak is calibrated at 1); IS is volume of the internal standard solution in pyridine added (0.1 mL); *M* is the molecular weight of the internal standard (386.65 g/mol); *L* is weight of the freeze-dried sample added in the vial (g); and *P* is the purity of the internal standard (0.99).

#### Molecular Weight Determination

The method used to determine the molecular weight of the lignosulfonates was based on the method published by Fredheim et al.,^29^ using size exclusion chromatography with UV detection (HPLC SEC-UV). The HPLC was fitted with a degasser, pump, autosampler, column oven, and UV detector. The eluent was run through the HPLC at analytical conditions for some hours for stabilization before standards and samples were injected. The system was calibrated using two broad lignosulfonate standards with known molecular weights previously determined using the above method published by Fredheim et al.^29^ to obtain absolute Mw and Mn data. The lignosulfonate samples and calibration standards are diluted with 2 mg of dry matter per 1 mL of eluent. Diluted samples and standards were injected from the vials, and the molecular weights were determined from the obtained chromatograms.^29^

### 2.4 Calcium Tolerance Test

Calcium tolerance (compatibility) tests were performed by mixing together oxidized lignosulfonate inhibitor solutions and calcium chloride solutions and afterward heating the mixed solution to 90 °C in a sealed jar. A matrix of tests was carried out with the inhibitor concentration in the mixture being either 100 or 1000 ppm and the calcium ion concentration being either 1000, 10000, or 30000 ppm. The solution was observed before heating and again after 1 h, 4 h, and 24 h at 90 °C to check for any occurrence of precipitates or turbidity. A clear solution throughout the test is an indication of good calcium compatibility for the oxidized lignosulfonates.

### 2.5 Thermal Stability Test

A 5 wt % solution of peroxide-treated oxy-LS4 in distilled water was placed inside a hard-glass tube fitted with a Teflon stopcock. The solution was then subjected to three repetitive cycles of vacuum refill (with nitrogen) before finally sealing off under a nitrogen atmosphere. The tube was then placed in an oil bath preheated at 130 °C and maintained at that temperature for 14 days. Afterward the solution was cooled and retested for calcite scale inhibition in the dynamic tube blocking rig.

### 2.6 High Pressure Dynamic Tube Blocking Scale Inhibition Tests

#### Preparation of Test Solutions

The synthetic brines used in this study were modeled according to the produced water from the Heidrun oilfield, Norway. For the calcite scaling, only formation water (FW) was used. The salt compositions and respective amounts for the Heidrun North Sea FW and the synthetic brines used in this study are listed in Table 2.

**Table 2:** Salt Compositions Used in the Scaling Tests

Ion	North Sea FW (mg/L)	Brine 1 (mg/L)	Brine 2 (mg/L)	Salts	Brine 1 (g/3L)	Brine 2 (g/3L)
Na^+^	19510	19510	19510	NaCl	148.77	148.77
Ca^2+^	1020	2040	0	CaCl_2_·2H_2_O	22.45	
Mg^2+^	265	530	0	MgCl_2_·6H_2_O	13.30	
K^+^	545	1090	0	KCl	6.23	
Ba^2+^	285	570	0	BaCl_2_·2H_2_O	3.04	
Sr^2+^	145	290	0	SrCl_2_·6H_2_O	2.65	
HCO_3_^–^	500	0	2000 (1000)	NaHCO_3_		8.26

#### Preparation of Cleaning Solution

Basic EDTA solution (pH ≈ 12) for cleaning out calcite scale from the scaling coil was prepared by dissolving approximately 120 g of Na_2_EDTA·2H_2_O and 40 g of NaOH in distilled water in 3 L of water.

Before each experiment, the brines, inhibitor solutions, and cleaning liquids are freshly prepared and degassed thoroughly to avoid complications during the automated run.

#### Test Protocol

**Figure 2:**
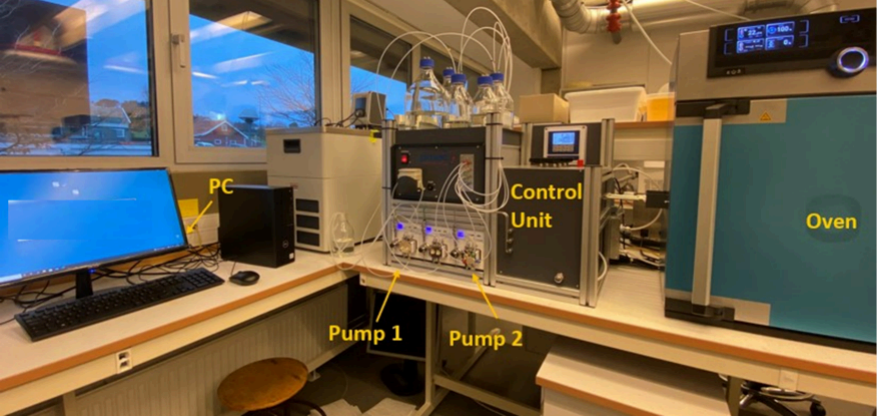
Two-pump dynamic tube blocking scale rig.

The scale inhibition study was performed on a high-pressure dynamic scale rig. The main dynamic tube blocking scale rig used in this study was manufactured by the PMAC Group, Aberdeen, UK (Figure 2).

The equipment has a main control unit consisting of two pumps that flow aqueous solution at a desired flow rate through a stainless-steel coil (1 m long and 1 mm of internal diameter). The coil is placed inside an oven, which is connected to the main control unit via steel tubing. The main control unit also contains a pH probe and a conductometer to measure pH and conductivity of the mixed aqueous solutions passing through the coil. The tests were carried out at 100 °C with a line pressure of ≈1200 psi.

**Figure 3:**
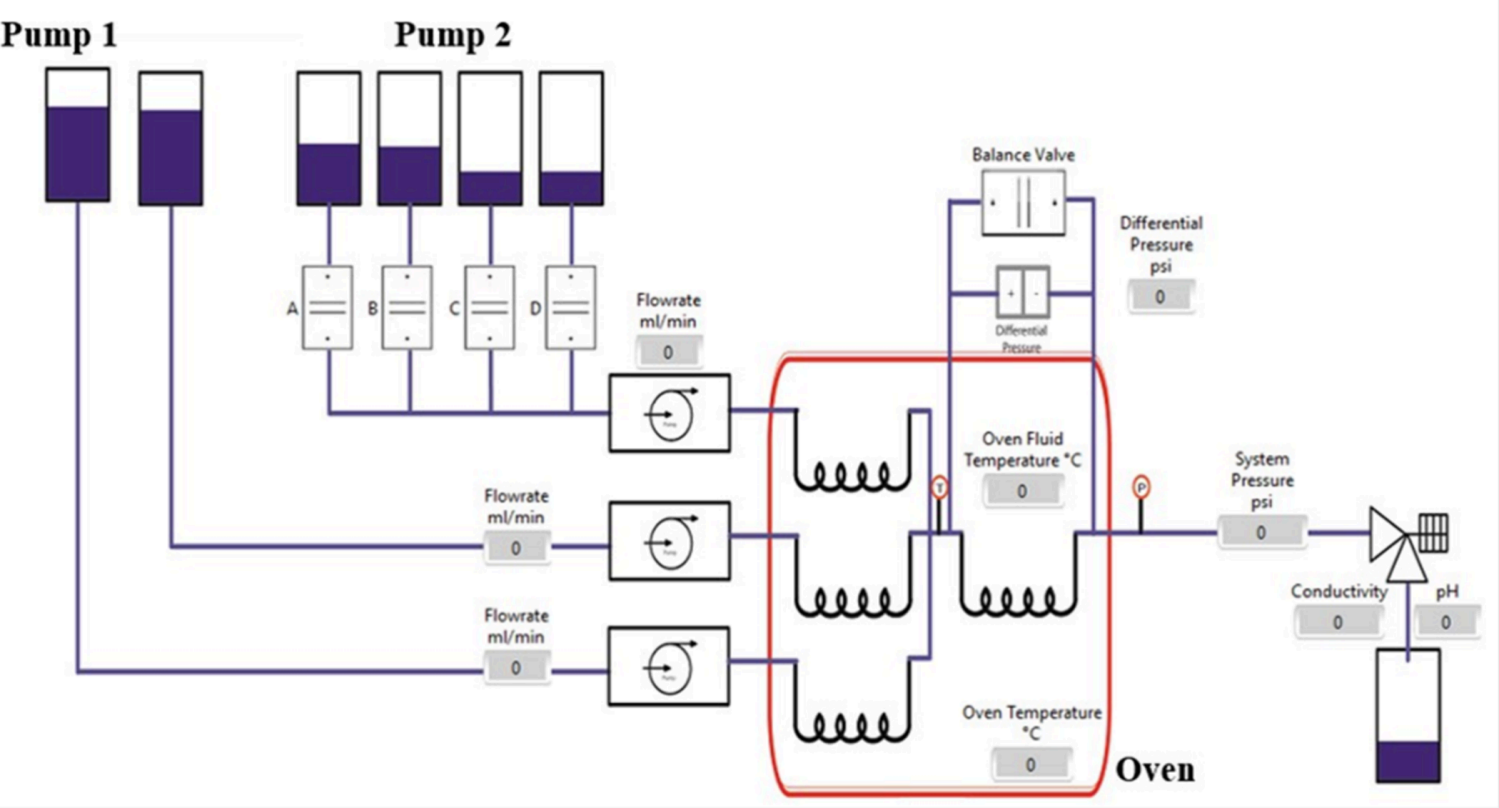
Schematic diagram of the two-pump dynamic tube blocking scale rig.

Figure 3 presents a schematic diagram of the control unit that explains a typical scenario during a one-tube blocking test run. Pump 1 injects cation brine aqueous solutions, while pump 2 is responsible to pump four different solutions controlled by valves A, B, C, and D. Valve A is responsible for pumping the anion brine aqueous solutions, and valve B injects inhibitor solution (dissolved in anion brine) at a certain flow rate which is then mixed and pumped into the scale coil. Valves A and B open and close simultaneously to maintain the flow rate inside the coil. In addition, valves C and D in pump 2 are responsible for injecting the cleaning solutions, basic EDTA solution (pH ≈ 12), and distilled water, respectively.

One full run consists of two successive tests controlled by the automated scale rig: The first test is “Chemical” where cation and anion brines are mixed with inhibitor solution and are pumped into the scaling coil at a flow rate of 10 mL/min. The test consisted of several automated periods, each with a duration of 1 h. In each test, the inhibitor concentration is decreased gradually until the rapid tube blocking occurs at a certain inhibitor concentration. The second test is “Repeat Chemical” which starts from a stage with inhibitor concentration the same as the one that led to rapid scale formation. The starting concentration of the stock inhibitor solution (inhibitor dissolved in anion brine) is about 200 ppm with pH ≈ 7. During the first “Chemical” stage of the test, the brines and the inhibitor solution are mixed at a certain ratio to obtain a desired inhibitor concentration inside the test coil. A 100 ppm initial inhibitor concentration is the starting point, and then every hour the automated rig adjusts the flow to have the concentration decrease gradually (e.g., 50, 20, 10, 5, 2, and 1 ppm) until rapid scale formation occurs, which then triggers the cleaning cycle.

**Figure 4:**
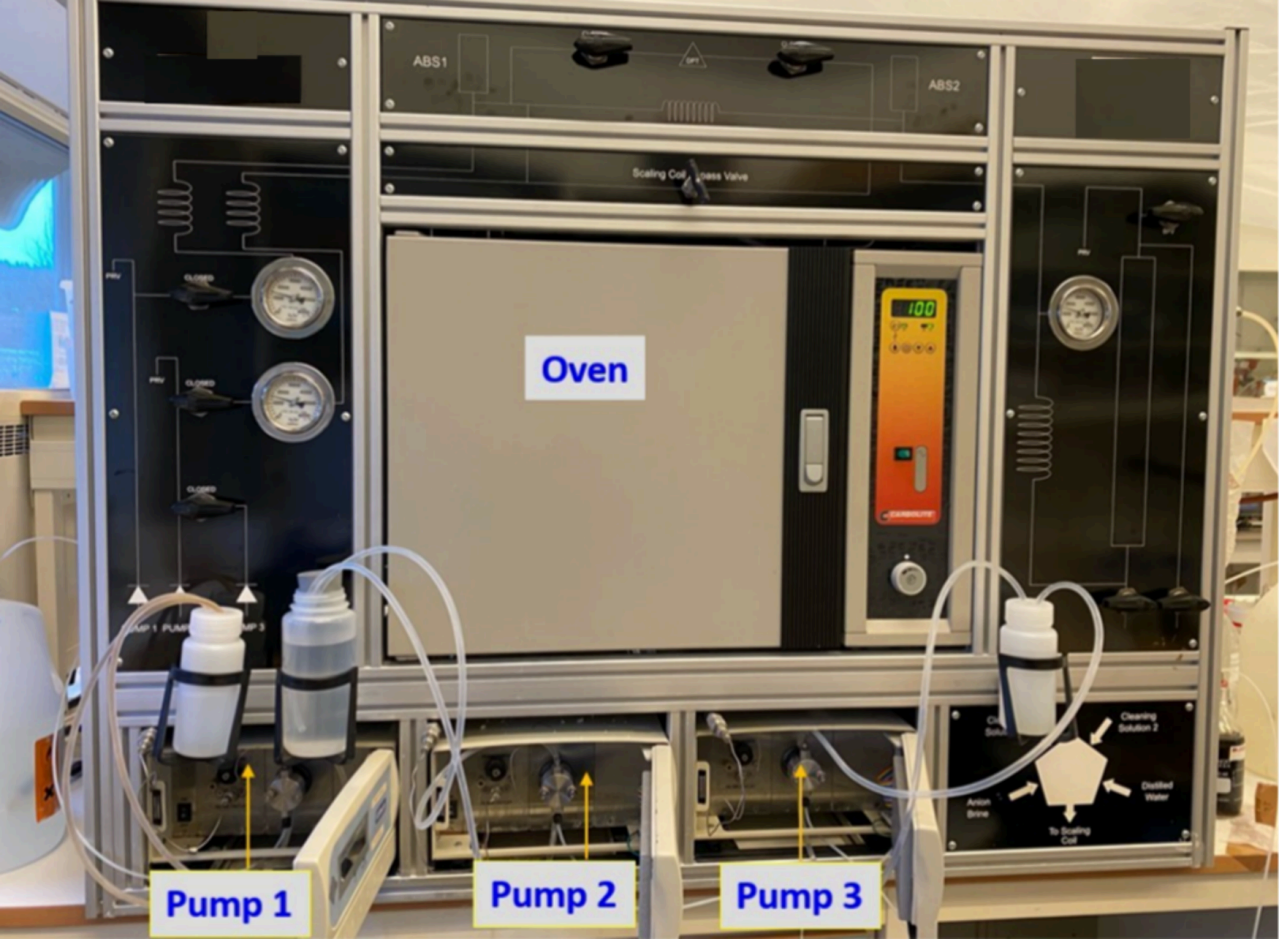
Picture of the triple-pump dynamic tube blocking scale rig.

The second dynamic tube blocking rig was manufactured by Scaled Solutions, Scotland (Figure 4). This rig was used in the early stages of this study, specifically for the untreated lignosulfonates. It functions in a way similar to that of the PMAC rig but does have a few differences in the design or the test settings. The Scaled Solutions rig has three pumps for pumping the liquids into the system and pump 1 and pump 2 for cation and anion brines, respectively, and pump 2 also pumps the cleaning solutions. Pump 3 is responsible for the inhibitor solution, which is usually an aqueous solution of the chemical with a concentration of 1000 ppm and pH ≈ 4–6. The scale coil is 3 m long; therefore, the baseline differential pressure is higher than the PMAC rig (3 psi vs 0.5 psi). A “blank test” with no chemical is performed in the start, together with the two “inhibitor tests”, during each run.

## 3 Results and Discussion

### 3.1 Ring-Opening Oxidation

Polycarboxylates are used for inhibiting a variety of scales, including carbonates (calcite, siderite) and sulfates (gypsum, celestine, barite), even at very low calcium concentrations. The anionic carboxylate groups (−COO^–^) can ionically bond to the divalent cations, mimicking and replacing the divalent anions such as carbonate (CO_3_^2–^) or sulfate (SO_4_^2–^) in the lattice structure.^1,3,11^ Several carboxylate interactions are necessary to enable a good interaction with the surface of the growing scale lattice. This is why polycarboxylates such as polyacrylates, polymaleates, and polyaspartates have found commercial use as scale inhibitors. The surface coverage of the polycarboxylate on the scale surface does not need to be very high (as low as 3–5%) in order to enable complete scale inhibition at the nucleation or crystal growth stage.^1^

**Scheme 1:**
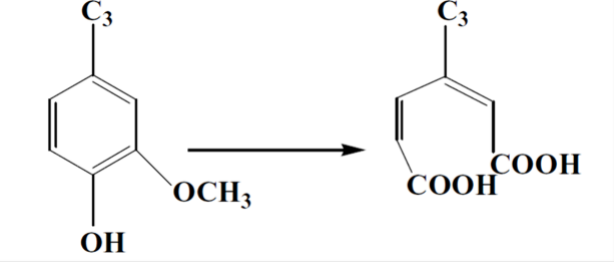
Idealized Reaction of Oxidative Ring-Opening Reaction on a Lignin Monomer Unit to Express Carboxylic Acids^31^

Lignin and lignosulfonates are predominantly methoxyphenolic in character and lack the high density of carboxylate groups that characterize many classes of polymeric scale inhibitors. This lack of active functionality explains why lignosulfonates are not generally used as scale inhibitors. One way to convert phenolic structures into carboxylic is through ring-opening oxidation reactions (Scheme 1, below), which has long been used to convert lignin into many different functional chemicals, from food flavorings to crystal growth retarders.^30,31^ However, oxidative lignin chemistry is challenging to control with many different reaction pathways. Oxidizing reactions must be selective toward ring opening in a way that expresses maximum density of carboxylate groups without significantly degrading the polymer. This requires a highly selective yet powerful oxidant. One such oxidant is peracetic acid (PAA), which has previously been reported to increase the carboxylate of lignin.^26^

Peracetic acid is a mixture mainly of hydrogen peroxide, acetic acid, and water along with some mineral acid. Usually in the presence of a strong mineral acid like HCl or H_2_SO_4_ the following equilibrium happens:
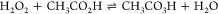
To prepare a peracetic acid reagent, we mixed hydrogen peroxide with acetic acid in the presence of a catalytic amount of mineral acid. The mixture was left at 4 °C overnight to equilibrate. The premixed peracetic acid was then added to an LS solution (∼25–30% w/w) at 70 °C. The peracetic acid oxidation reaction was performed under different conditions, varying reaction pH by adjusting with NaOH or mineral acid, and at different peroxide:acetic acid ratios (Table 2). The reaction product was then measured for scale inhibition to determine the fail inhibitor concentration (FIC) value in the dynamic tube blocking test.

### 3.2 Calcite Scale Inhibition Performance

The inhibition performances of the oxidized lignosulfonates were compared with those of the unreacted LS starting material and a commercial polycarboxylate benchmark inhibitor (Table 2) using a high-pressure dynamic tube blocking scale method. Brine composition, pH, temperature, and pressure were chosen to test against the calcium carbonate scale under conditions that mimic the water produced in Heidrun oilfield, Norway. The fail inhibition concentration (FIC) corresponds to the concentration of inhibitor at which scale starts to form when reducing the inhibitor concentration from 100 to 2 ppm. A lower FIC shows that less inhibitor is required to inhibit the scale and therefore has better performance.

The calcite scale inhibition results in Table 3 show that the synthetic polycarboxylate benchmark inhibitor has an FIC of 5 ppm. This is significantly lower than the unreacted LS, which is not effective at the inhibiting scale even at the maximum concentration at the start of the test (FIC = 100 ppm). Reaction of the LS with peroxide without acetic acid (1:0 ratio) did not improve the scale inhibition performance. However, when reacted with the peracetic acid mixture, the FIC value of the lignosulfonate drops from 100 ppm to 5–10 ppm. Optimal performance was achieved at low pH, and the peroxide:acetic acid ratio and type of mineral acid catalyst did not have a significant impact on FIC. This may indicate that the selectivity of the peracetic acid oxidation reaction toward the active product is mainly controlled by pH.

**Table 3:** Calcite Scale Inhibition Performance at 100 °C of the Lignosulfonate Start Material, Oxidized Lignosulfonates, and Benchmark Polycarboxylate Inhibitor

Reaction conditions (PAA composition, pH etc.)	FIC value after oxidation (ppm, min)
Benchmark: Co-MA:AA (copolymer maleate:acrylate)	5 ppm, 21 min
Unreacted lignosulfonate	100 ppm, 28 min
H_2_O_2_:AcOH, 1:0 v/v	100 ppm, 28 min
H_2_O_2_:AcOH, 4:1 v/v + 0.5 mL HCl, reaction pH ≈ 1.5	5 ppm, 28 min
H_2_O_2_:AcOH, 4:1 v/v + 2 mL H_2_SO_4_, reaction pH ≈ 1.5	5 ppm, 25 min
H_2_O_2_:AcOH, 4:1 v/v + 2 mL H_2_SO_4_, reaction pH ≈ 6–7	10 ppm, 30 min
H_2_O_2_:AcOH, 4:1 v/v + 2 mL H_2_SO_4_, reaction pH ≈ 4	10 ppm, 28 min
H_2_O_2_:AcOH, 3:1 v/v, 2 mL H_2_SO_4_ (50%), reaction pH ≈ 4–5	10 ppm, 25 min

The reason for the improved performance when synthesizing the oxidized lignosulfonates at low pH is because the formation of peracetic acid is promoted at low pH. At low pH, more of the peroxide converts to the peracetic acid, which is more selective to the ring-opening reaction proposed as the favorable pathway to carboxylate formation. Therefore, low pH promotes the ring-opening carboxylate formation oxidative reaction that leads to a higher carboxylate density and a more efficient inhibitor. At high pH the decomposition of peracetic acid to acetic acid and peroxide is accelerated.

### 3.3 Calcium and Temperature Tolerance

Scale formation in oilfield applications typically happens under challenging conditions, with high concentrations of multivalent metal ions (especially calcium) in the brine and high temperatures. Polymeric scale inhibitors often degrade or precipitate under such conditions, reducing the inhibition performance and causing problems with deposits.

**Figure 5:**
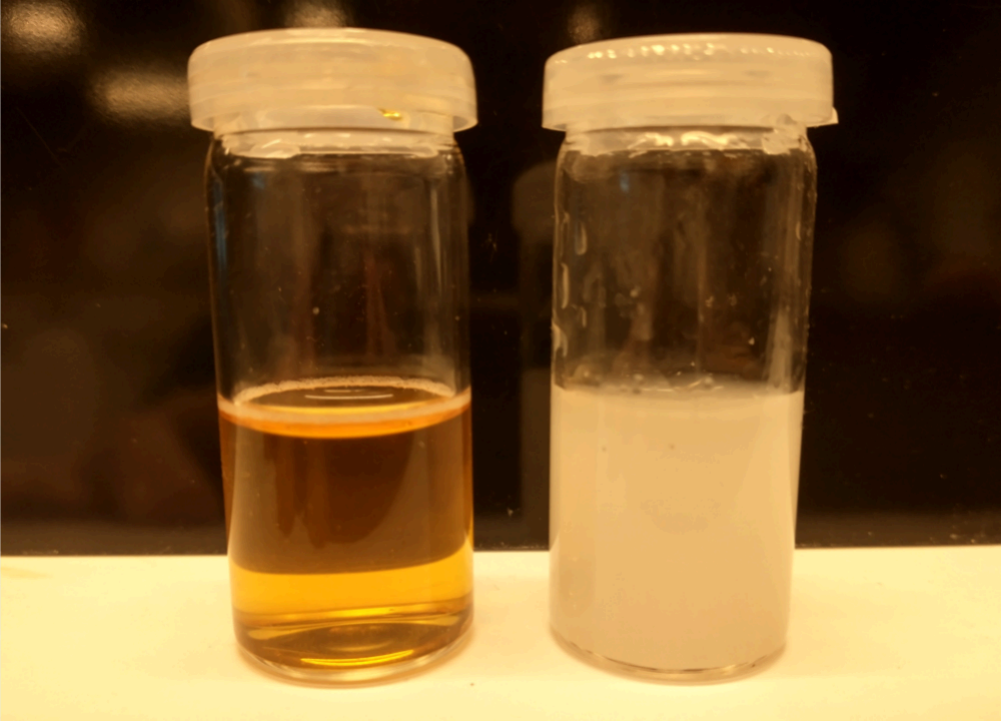
1000 ppm of oxidized lignosulfonate (left) and 1000 ppm of polycarboxylate (right) in 1000 ppm of calcium ions after 1 h at 90 °C.

**Figure 6:**
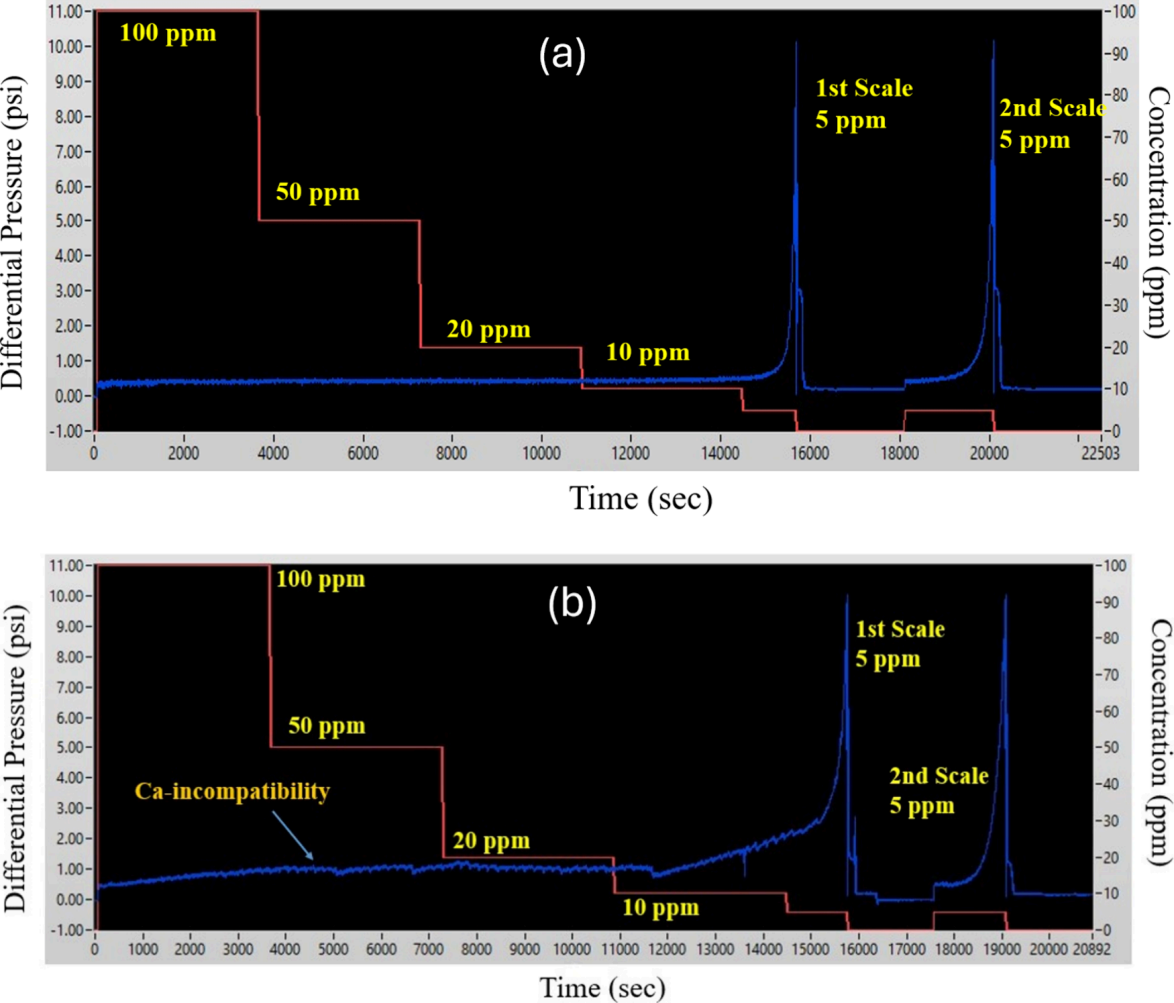
Schematic representation of the differential pressure data at a stepwise decreasing concentration of inhibitor concentration. Peracetic acid oxidized LS (a, top) compared with the commercial polycarboxylate benchmark (b, bottom). Note the undulating baseline and increase in the differential pressure data for the polycarboxylate before calcite scale formation, indicating calcium intolerance.

Simple solubility tests in concentrated calcium chloride brines were employed to determine the calcium tolerance of the oxidized LS. The inhibitor polymer was dissolved in the brine, and the samples were analyzed for turbidity. Tolerance to the high calcium brine is indicated by the absence of turbidity, as shown in the left vial in the photograph in Figure 5. The brine containing the oxidized LS was clear (indicating salt tolerance), whereas the brine containing the poly(carboxylate) benchmark was turbid (indicating salt intolerance). In this study, the oxidized lignosulfonates showed excellent compatibility, even for 10000 ppm of LS and 30000 ppm of Ca^2+^ ions. The oxidized LS shows much superior calcium compatibility to polycarboxylates such as polyacrylates.^32−36^ This difference in calcium tolerance is also evident in the differential pressure data from the tube blocking test, indicated by the flat baseline for the oxidized lignosulfonate (Figure 6a) compared to the undulating baseline for the poly(carboxylate) (Figure 6b).

**Figure 7:**
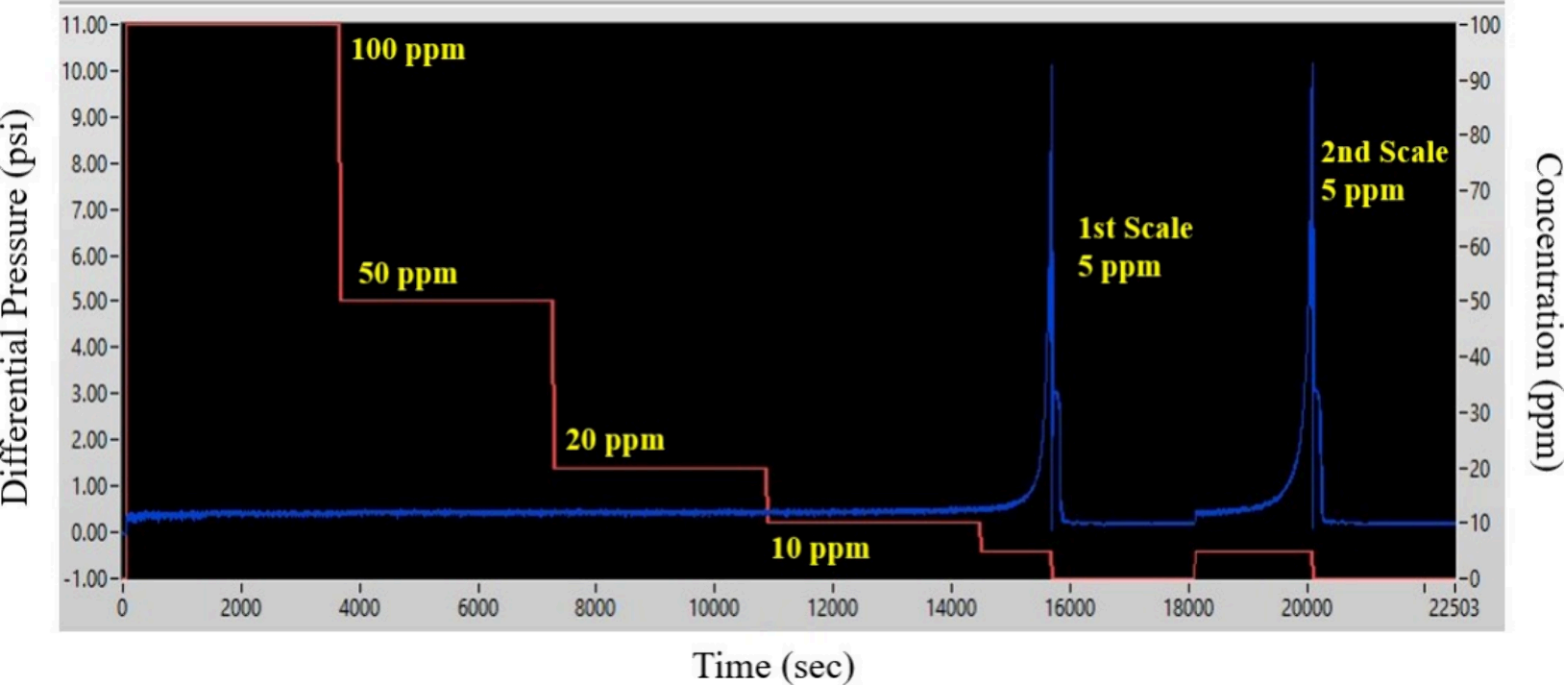
Schematic representation of the differential pressure data at stepwise decreasing concentration of oxidized LS after 130 °C high-temperature treatment. Note the flat baseline and 5 ppm FIC peaks that closely resemble the data from oxidized LS without thermal treatment (Figure 6a).

Temperature tolerance tests were conducted by storing a 1% oxidized lignosulfonate (F) solution for 10–14 days at 130 and 160 °C and then remeasuring the FIC value (Table 4). A large increase in FIC is indicative of temperature intolerance from thermal degradation. No change in FIC value was determined after 130 °C storage (Figure 7), and only a small increase from 5 to 10 ppm FIC was determined after 160 °C storage. This shows that the oxidized LS has an excellent temperature tolerance.

**Table 4:** Performance of Lignosulfonate F after Thermal Treatment

Oxidized sulfonated lignin	FIC before thermal treatment^a^	FIC after 14 days at 130 °C^a^	FIC after 10 days at 160 °C^a^
F	5 ppm	5 ppm	10 ppm

a Determined according to the scale rig test described below.

### 3.4 Structural Analysis

Plants evolved lignin to have a highly random structure, which resists microbial attack. The macromolecular structure of lignin is probably randomly branched, made up of random combinations of various repeating methoxyphenolic monomeric units linked through different structures. This fundamental structural feature of native lignin, along with the various coproducts and polydispersity of industrially produced lignin materials, makes accurate structural determination extremely challenging. An unambiguous structural model of lignin remains elusive and continues to be the subject of cutting analytical research,^37^ but analytical techniques have been developed to indicate contents of specific functional groups.

The central hypothesis of this study was that ring-opening oxidation would transform the inactive methoxyphenolic lignin backbone structure into an active scale, inhibiting carboxylic structure.^38^ As illustrated in Scheme 1, the desired reaction gives an increase in carboxylic groups with a simultaneous decrease in methoxy groups. Determining the content of such groups on the lignin backbone is challenging because of the occurrence of low MW coproducts and reagents (e.g., acetic acid) that also contain carboxylic moieties. Therefore, the lignosulfonate products were treated to isolate the lignosulfonate and remove low MW inactive coproducts, such as acetic and formic acids. The purified lignosulfonate material was then analyzed for carboxylic acid, sulfonate, and methoxy group content.

Table 5 shows the results from the analytical analysis of the lignosulfonate starting material compared to the oxidized lignosulfonate. Consistent with the hypothesis, the peracetic acid oxidation significantly reduces the methoxy content and increases the carboxylic content. The sulfonate content of the oxidized LS, expressed as % organic sulfur, remains unchanged during the oxidation. This is advantageous as sulfonate groups are known to actively inhibit scale and improve salt tolerance and are indicative of the selectivity of the reaction toward ring opening. Oxidation resulted in only a modest decrease in molecular weight, showing that the reaction was selective and did not result in general degradation of the polymer.

**Table 5:** Performance and Molecular Characteristics of Untreated and Oxidized LS

Sulfonated lignin	MW (kDa)	Mn (kDa)	Methoxy (%)	COOH (%)	Organic Sulfur (%)	Calcite FIC (ppm)	Calcium compatibility
LS	30	3	9	1	6	100	Good
Oxidized LS	24	4	5	5	6	5	Good

## 4 Conclusion

Several commercial lignosulfonates performed relatively poorly as calcite scale inhibitors in dynamic tube blocking tests. However, oxidized lignosulfonates, which can be thought of as polycarboxylates, show much improved inhibition effect. Lignosulfonates that gave FIC values of 50–100 ppm could be oxidized to give FIC values of 5 ppm. The oxidized lignosulfonates were made in a one-step reaction with readily available ingredients, hydrogen peroxide and acetic acid, giving peracetic acid in situ.

The oxidized lignosulfonates also showed excellent calcium compatibility even with 30000 ppm calcium ions at 90 °C. Oxidized lignosulfonates also gave thermal stability, giving no loss of performance after aging at 130 °C (FIC = 5 ppm) and only a small loss when aged at 160 °C (FIC = 10 ppm). These properties are useful for downhole squeeze treatments also in high-temperature wells. This present study unequivocally establishes oxidized lignosulfonates as a new class of sustainable green scale inhibitor, thereby bridging the gap between materials derived directly from nature and the classic, synthetic polymeric scale inhibitors.
